# Osteochondritis dissecans of the metacarpal head in a soldier treated with osteochondral autograft transplantation surgery: A case report

**DOI:** 10.1097/MD.0000000000032563

**Published:** 2023-01-20

**Authors:** Dong-Geun Kang, Dong Hyun Lee, Jin-Hyung Im

**Affiliations:** a Department of Orthopedic Surgery, Gyeongsang National University, College of Medicine and Gyeongsang National University Changwon Hospital, Changwon-si, Gyeongsangnam-do, Republic of Korea; b Department of Orthopedic Surgery, Yeouido St. Mary’s Hospital, College of Medicine, The Catholic University of Korea, Seoul, Republic of Korea.

**Keywords:** case report, hand, osteochondral autograft transplantation surgery (OATS), osteochondritis dissecans (OCD), subsidence

## Abstract

Osteochondral autograft transplantation surgery (OATS) was introduced as a treatment for hand osteochondritis dissecans (OCD), and successful results were reported. However, the cartilage height of the donor plug has not yet been established compared with that of other joints. We present a case using OATS for OCD of the 3rd metacarpal (MCP) head that occurred in a soldier and the findings of magnetic resonance imaging (MRI) images taken at 12 weeks after surgery.

**Patient concerns:**

A 21-year-old male presented with a painful, swollen 3rd MCP joint in the right hand. He was a soldier and right-handed; in addition, he did not recall any traumatic events, nor had he taken any corticosteroid medications before. The patient was excluded from military training and underwent conservative treatment with medication and a brace in a military hospital for 3 months.

**Diagnosis:**

The patient was diagnosed with OCD due to avascular necrosis of the 3rd MCP head of the right hand through X-ray and MRI.

**Interventions:**

OATS was planned as a surgical treatment. Surgery uncovered a 10 × 10 mm^2^, isolated cartilage defect of the 3rd MCP head that had an irregular margin and a loose body. Using Arthrex OATS, a 10-mm diameter, 10-mm depth hole was made at the articular defect site, and an 11-mm diameter, 12-length plug was harvested from the left lateral femoral condyle. The donor plug was inserted into the prepared defect site with press-fit fixation.

**Outcomes:**

At the last follow-up, the height of the articular cartilage had subsided with union on MRI 12 weeks after the surgery. However, the patient was asymptomatic with a normal range of motion of the right hand, and he returned to military training.

**Lessons:**

Although the joints of the hand are small and non-weight bearing, the level of articular cartilage of the donor plug was subsided in a follow-up MRI in our case. We suggest that the difference in cartilage thickness between the donor and the recipient might have been the cause of subsidence of the articular cartilage, and therefore, it may be helpful to transplant donors with similar thicknesses of articular cartilage.

## 1. Introduction

Osteochondritis dissecans (OCD) is a joint cartilage disease that was identified by KK¨onig in 1887.^[1]^ It is caused by a subchondral bone blood flow decrease associated with prior fracture, repetitive trauma, vasculitis, autoimmune disorder, or idiopathic etiology, and it results in instability and disruption of articular cartilage that causes debilitating pain and dysfunction of the joints involved and progresses to degenerative osteoarthritic changes.^[2]^ Because OCD rarely occurs in the hand joint but mainly occurs in the knee, elbow joint and talus, a treatment algorithm for the hand has not yet been established as it has for the knee, elbow joint and talus. Cartilage implantation and osteochondral autograft transplantation surgery (OATS) were introduced as the treatments for hand OCD, and successful results were reported. We present a case using OATS for OCD of the 3rd metacarpal (MCP) head that occurred in a soldier without a history of trauma.

## 2. Patient information

A 21-year-old male presented at the outpatient department with a painful, swollen 3rd MCP joint in the right hand. He was a soldier and right-handed; moreover, he did not recall any traumatic event, nor had he taken any corticosteroid medication. He was excluded from military training and underwent conservative treatment with medication and a brace at a military hospital for 3 months.

## 3. Clinical findings

No signs of infection were observed. The range of motion of the right middle finger was limited, and pain was aggravated in full extension. The patient could not make a tight fist or lift anything heavy and, consequently, could not carry out military exercises.

## 4. Diagnostic assessment

**Figure 1.**
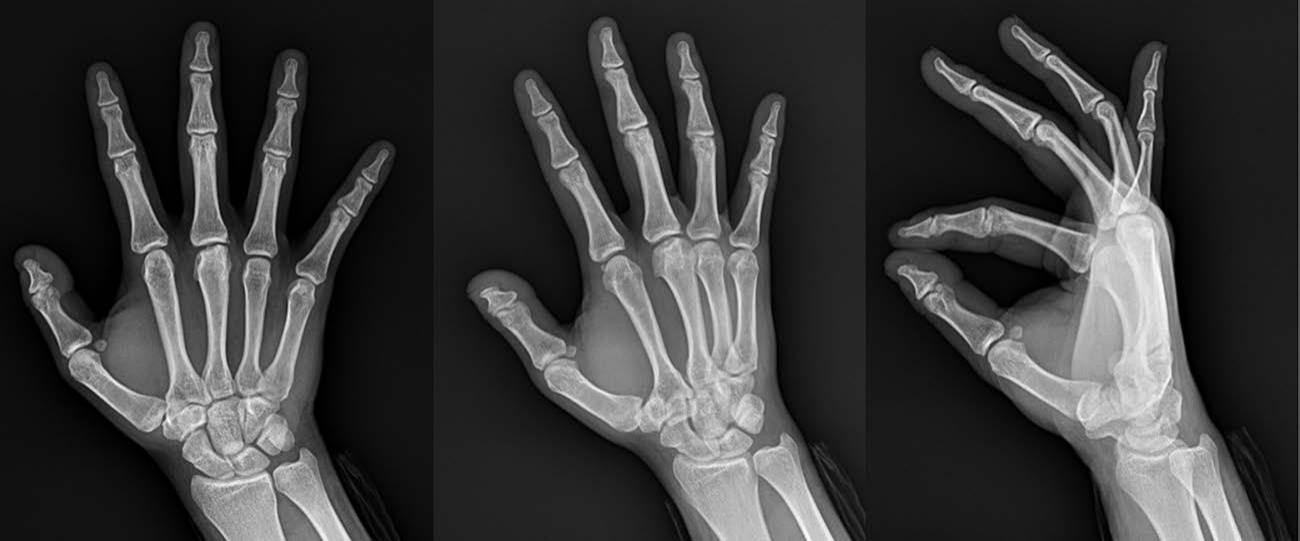
Anteroposterior view of the right-hand middle finger MCP joint showing a flattening articular fracture of the metacarpal head. MCP = metacarpal.

**Figure 2.**
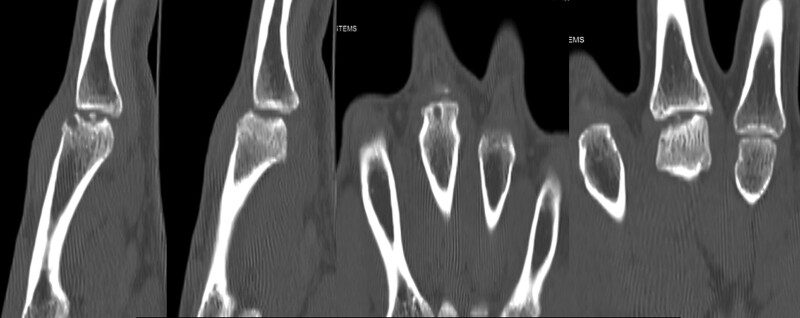
Computed tomography scans of the right hand demonstrating cystic change, fragmentation of the 3rd metacarpal head.

**Figure 3.**
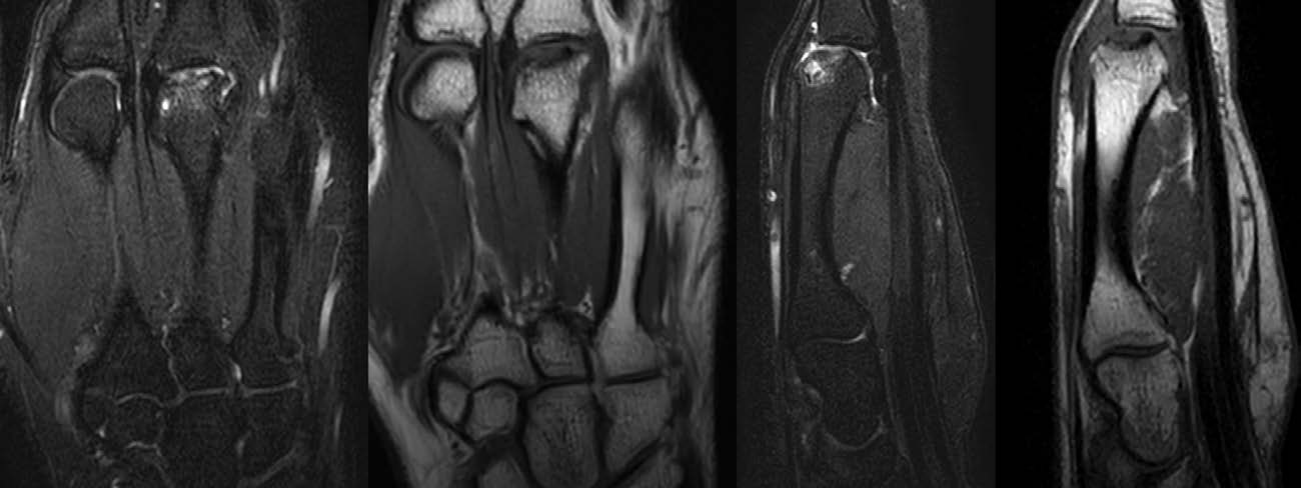
Magnetic resonance image scans of the right hand demonstrating articular cartilage wear, subchondral edema and signal change consistent with avascular necrosis of the 3rd metacarpal head.

The white blood cell count, erythrocyte sedimentation rate, C-reactive protein, and coagulation panel results were normal in the laboratory exam. On X-ray images, an irregular margin at the 3rd MCP head was observed, and more than two-thirds of the joint surface was involved (Fig. 1). Subchondral bone defects of the 3rd MCP head and loose bodies in the joint were observed on computed tomography (Fig. 2), and destruction of joint cartilage and changes in subchondral bone of the 3rd MCP head were observed on magnetic resonance imaging (MRI) (Fig. 3).

## 5. Therapeutic intervention

**Figure 4.**
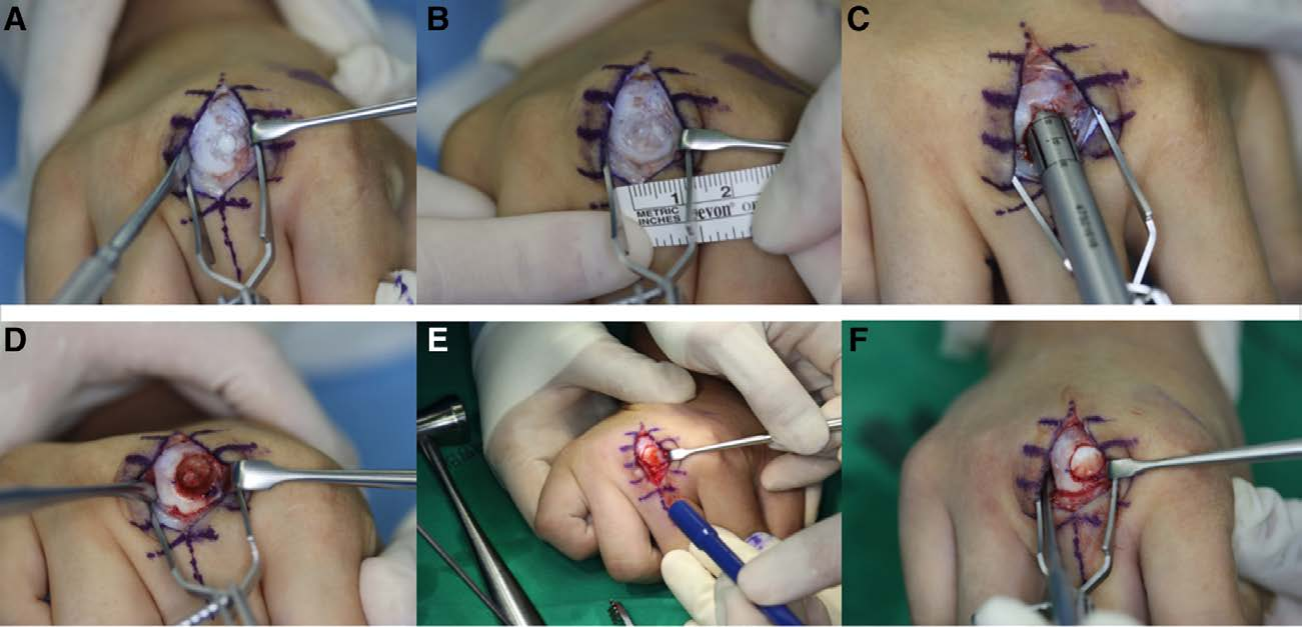
(A) A longitudinal capsulotomy was made for the entire 3rd MC head to provide adequate exposure. An articular cartilage destruction and osteochondral fragmentations were observed. (b) After debridement of joint, the defect size was measured. (C) A 10 mm-sized recipient chisel was inserted at a depth of 10 mm over the defect. (D) A recipient hole with a diameter and depth of 10 mm was prepared. (E) After the delivery of the donor plug, a careful cartilage leveling was performed by gentle press fit tamping. (F) The congruency of articular cartilage was confirmed. MC = metacarpal.

**Figure 5.**
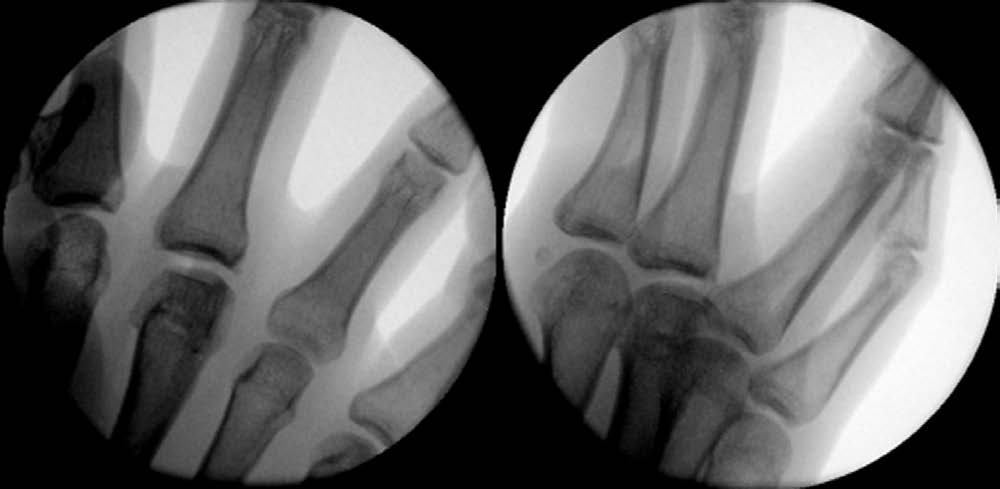
A proper placement of the donor plug was confirmed and the 3rd MCP joint ranged smoothly under the fluoroscopic images. MCP = metacarpal.

Surgical treatment was considered, and OATS from the non-weight bearing portion of the distal femur was planned. The Arthrex OATS system (Arthrex, Naples, FL), which includes single-use donor and recipient cutting tube sets, was used. The patient was positioned supine with the upper limb resting on a hand table, and tourniquets were placed on both the right upper arm and the left thigh. After inflation of the tourniquet on the upper arm, a midline skin incision was created, and the extensor digitorum communis was separated in half to expose the MCP head. Loose bodies and isolated cartilage defects were observed, and the size of the defect was measured (Fig. 4A and B). Under fluoroscopy, the location of a Kirschner wire and the trajectory of the chisel were identified to avoid cortical penetration of the chisel, and a 10 mm-sized recipient chisel was inserted at a depth of 10 mm over the defect (Fig. 4C and D). After inflation of the left thigh tourniquet, the non-weight bearing portion of the lateral femoral condyle between the sulcus terminalis and the physis was exposed through a lateral parapatellar approach. A portion similar to the contour of the MCP defect was identified and harvested using a donor chisel. The diameter of the donor plug was 1 mm larger than the defect using the donor chisel designed diameter, and the depth was 2 mm larger than the recipient plug’s depth of 10 mm. The knee wound was closed in layers after harvest of the donor plug, and the tourniquet was deflated. The donor plug was inserted into the recipient hole of the MCP, paying attention to the orientation for correct articular contour. Gentle tamping was followed to match the level with the surrounding cartilage (Fig. 4E and F). Proper placement was confirmed using fluoroscopy, and the 3rd MCP joint ranged smoothly (Fig. 5). The hand wound was closed, including the extensor digitorum communis, and the hand was immobilized in the intrinsic plus using a short arm splint.

## 6. Follow-up and outcomes

**Figure 6.**
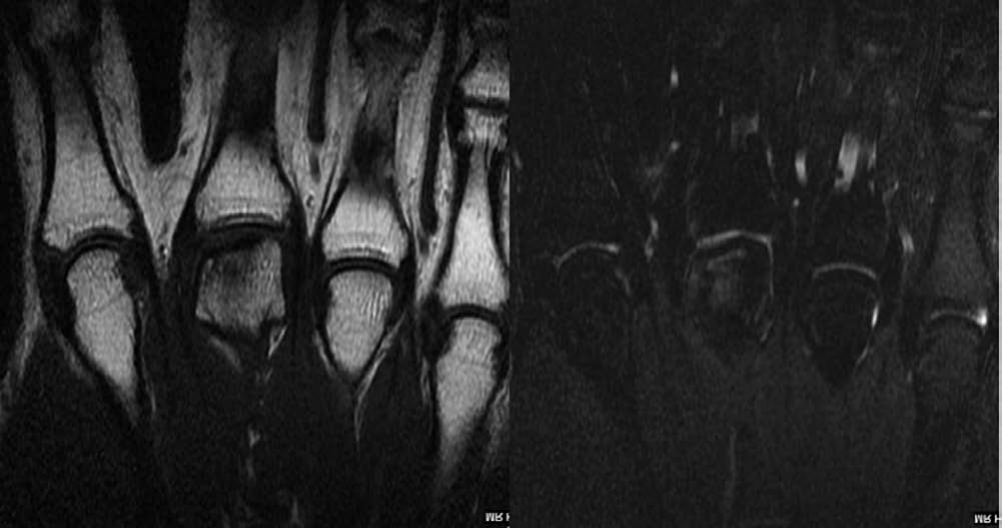
T1 weighted image showing bony union of the plug and T2 weighted image showing articular cartilage subsidence of the 3rd metacarpal head on magnetic resonance image at 12 weeks after the surgery.

Ambulation with weight bearing began the day after the surgery, and rehabilitation for the left knee was not needed. The MCP joint exercises started 4 weeks after the surgery and ranged from active motion to passive motion with a removable brace. The brace was worn up to 2 months after the surgery. Approximately 9 weeks after the surgery, the range of motion of the MCP joint was normal without pain, and union of the donor plug was observed on the radiographs. The patient returned to military training approximately 12 weeks after the surgery, at which time, bony union of the plug articular and cartilage subsidence of the 3rd MCP head was observed on MRI (Fig. 6).

## 7. Discussion

Osteonecrosis of the MCP head was first described by Dieterich in 1932.^[3]^ A natural history has not been well delineated or adequately described because the disease is rare and most reports are of single cases; moreover, osteonecrosis of the MCP head has been associated with trauma and corticosteroid medication, but it may also develop without an obvious etiological factor, and it is then termed “‘idiopathic’.”^[4–7]^ Any MCP can be affected, and the pathological changes are similar to those described in other sites, such as the femur. Several studies have implicated a hypercoagulable state as a potential cause of osteonecrosis in the proximal part of the femur.^[3,8]^ The present patient did not have any trauma history, medication history, or underlying disease. We hypothesize that military training might be correlated with OCD. To the best of our knowledge, OCD has been reported frequently in baseball players and athletes, but there have been no reports of nontraumatic events in the military.

The OATS procedure is commonly used for resurfacing OCD of the knee, ankle, and elbow joints.^[9–13]^ Whereas in the short term, pain alleviation is important, in the long term, grafting OCDs with autologous hyaline cartilage may prevent osteoarthritis. In the OCD of hand MCP joints, OATS from the femoral condyle would provide an adequate articular surface for contact compared with a less desirable MCP fusion. Graft stability is thought to be provided by press-fitting grafts into smaller recipient holes.^[14,15]^ However, postoperative graft subsidence has been reported in 10% to 17% of patients.^[16–19]^ Subsidence leads to increased rim stress in adjacent cartilage (14) and fibrocartilaginous graft overgrowth.^[20,21]^ Although subsidence might occur after OCD, Huang FS et al reported that hyaline articular cartilage thickened with cellular hypertrophy and that the 1 mm countersunk was compensated for by cartilage hypertrophy in the knee joint at 6 weeks after surgery in a sheep model.^[21]^ İskender Ö et al reported that the cartilage thickness of MCP heads is thicker in weight lifters and volleyball players than in healthy control groups due to repetitive load on the MCP head in these athletes. In the present case, even though the level of cartilage of the donor plug was in flush placement with bottomed insertion and enough time had elapsed for articular cellular hypertrophy to occur, a slight subsidence was observed in the 12-month follow-up MRI. This is believed to occur during the joining of the plug bone to the surrounding bone. However, it is difficult to anticipate how much the cartilage level will sink and to insert the donor plug at sufficient elevation to prevent subsidence at the time of surgery. Furthermore, joint contact pressure may increase if the cartilage height remains elevated. A finite element analysis study reported that contact pressure does not increase significantly at 1 mm subsidence in the ankle joint,^[22]^ but there have been few studies on non-weight bearing and small joints. Therefore, it seems necessary to use a part with a similar cartilage thickness as a donor. Many studies have evaluated donor sites with similar cartilage thicknesses and contours for other joints because there is a difference in biology and composition according to cartilage thickness.^[23–27]^ Although there are few studies on the appropriate donor for the MCP head, the metatarsal head^[28]^ and a nonarticular part of the affected MCP^[29]^ were used as donors in OCD of the MCP head. However, revision surgery, such as removal of the fixation device, was needed, and a decreased range of motion was reported. OCD of the hand and wrist was treated using OATS without a fixation device and with the femoral condyle used as a donor.^[30]^ Although successful functional and radiological outcomes were reported, only bony union of the donor was observed, and there was no observation of articular height subsidence and no follow-up MRI evaluation. The MRI evaluation of the present case showed articular height subsidence at the 12-month follow-up. There are few studies on MCP head cartilage thickness, and a study of MCP head cartilage thickness using ultrasound measurement reported a value of 0.09 mm.^[31]^ It thus seems necessary to find a donor with a similar cartilage thickness and radius and less morbidity.

In conclusion, although OATS is a good treatment option in OCD of the MCP head, more studies regarding a similar cartilage thickness and curvature according to the recipient site are needed in the future.

## Acknowledgment

This article was edited by a professional English language editing service, Nature Research Editing Service.

## Author contributions

**Data curation:** Dong Hyun Lee.

**Formal analysis:** Dong Hyun Lee.

**Writing – original draft:** Dong-Geun Kang.

**Writing – review & editing:** Jin-Hyung Im.
